# What Would Jenner and Pasteur Have Done About COVID-19 Coronavirus? The Urges of a Vaccinologist

**DOI:** 10.3389/fimmu.2020.02173

**Published:** 2020-08-26

**Authors:** Clarisa B. Palatnik-de-Sousa

**Affiliations:** ^1^Institute of Microbiology Paulo de Góes, Federal University of Rio de Janeiro (UFRJ), Rio de Janeiro, Brazil; ^2^Institute for Research in Immunology, Faculty of Medicine, University of São Paulo (USP), São Paulo, Brazil

**Keywords:** COVID-19, SARS-CoV2, inactivated virus vaccines, QS21 saponins, universal vaccine

## Introduction

Vaccines are the best cost-benefit tools to control and eradicate infectious diseases. The live smallpox vaccination, called variolation, was the injection of the homologous virus and this promoted self-healing local lesions that guaranteed strong and long-lasting protection. However, since 3% of these variolations caused cases of smallpox in the vaccinated individuals, it was considered unsafe and was discontinued (1–5).

In 1796, Edward Jenner, who had been variolated, discovered the vaccination principle when he used the cowpox virus live-vaccine (vaccinia virus) to induce cross-immunity and prevent human smallpox in a child. His strong merit was to initiate the campaign that turned vaccination against smallpox obligatory and universal, and to discover that cross-protection promoted by a heterologous, although related, organism was sufficient to guarantee efficacy and reduce the safety issues of the homologous live-vaccine (6).

In 1967, due to the World Health campaigns, smallpox was considered the first and only human viral infection ever eradicated (4, 5). Ironically, Jenner never knew that smallpox was induced by a virus (6, 7) which suggests that, what is needed for the eradication of a disease is the systematic worldwide use of a potent and efficient vaccine.

After the Sabin anti-polio vaccine, which was launched in the 1960's, many other vaccines have been developed based on whole attenuated viruses. However, poliomyelitis was also induced by the Sabin vaccine poliovirus 2 in healthy subjects (8–10). This means that, nowadays, live-vaccination with whole wild or attenuated virus is no longer ethically possible, mainly because of the large population of immunocompromised subjects, in which a live-vaccine could cause the disease. Due to these safety issues, whole virus and bacterial dead or inactivated vaccines have progressively substituted live-vaccines.

We could guess that this is precisely what Louis Pasteur, the father of Microbiology, would have done to control and eradicate COVID-19. Although he worked initially with the attenuation of viruses and bacteria, after his successful work with Rabies, fowl cholera and Anthrax (11), it became clear three steps were needed to develop a protective vaccine against infection. First, the organism should be isolated, then inactivated, and finally injected (5). In 1885, Pasteur's rabies vaccine employed an air dried fixed virus. Semple improved the fixation by adding phenol (12). Currently β-propiolactone is considered to be better than phenol or formaldehyde. However, it is carcinogenic (13). Therefore, other methods like ultraviolet or gamma-irradiation, high pressure (14), visible ultrashort pulsed laser, and low-energy electron irradiation have been suggested (15).

## Advantages of the Whole Inactivated Virus Vaccine

The most important advantage of whole inactivated vaccines is that, unlike the live or attenuated vaccines, they do not cause the disease (Table 1). In fact, inactivated vaccines preserve the intact structure of the antigens and their B-cell epitopes that enable them to interact with the antibody paratopes, and promote the synthesis of neutralizing antibodies. They can not only stimulate the humoral, but the cellular immune responses as well, in a manner similar to live viruses, since they preserve the virus structures during inactivation (15). Cross-presentation of conserved epitopes to the cytotoxic T cells (CTL), through the major HLA class 1 histocompatibility system, in addition to the viral pathogen associated patterns (PAMPS), which use innate immune receptors such as Toll like receptor 7, can induce T cell mediated responses. In fact, in the late endosome of the infected APC three different events can occur: (1) viral degradation following the exogenous pattern and presentation to CD4 T cells via the MHC class II molecules, (2) cross presentation pathway to CD8 T cells via the MHC class I molecules, and (3) viral membrane fusion following the endogenous pathway. The above mentioned pathways along with the recognition of viral PAMPS, using PRRs such as TLR7, as well as production of cytokines such as IFN-1 can promote potent cellular mediated immune responses (15). However, in the case of Influenza vaccines for instance, the inactivated formulations may not always induce T cell responses as potent as the live-vaccines. In fact, the inactivated vaccine may even prevent or suppress the induction of cross-reactive CD8^+^ T-cells (16).

**Table 1:** Evolution of vaccine compositions.

**Types of vaccines**	**Live or attenuated**	**Inactivated**	**Exotoxins**	**Recombinant and DNA vaccines**
Neutralizing antibodies	Yes	Yes	Yes	No
Reversion to virulence	Yes	No	No	No
Protection	High	Low	Low	No
CMI (TH1)	Strong	Yes	Yes	No
CTL (CD8)	Strong	Yes	Yes	No
Disease in immunosuppressed	Yes	No	No	No
Contain DNA, LPS, or other PAMPS	Yes	Yes	Yes	No
Requires adjuvants	No	No	No	Yes

PAMPS are constitutive components of the virus and bacteria (viral or bacterial nucleic acids, polysaccharides, Lipopolysaccharides, Lipid A, monophosphoryl lipid A, bacterial peptidoglycans, etc.). They are compounds universally recognized by the innate immune system of healthy subjects, who build a natural protective response against them. In contrast, modern purified, fractionated, recombinant, or synthetic vaccines gained in safety but lost potency because they lack the PAMPS (Table 1). While, vaccines using inactivated organisms with PAMPS have shown great success against polio, whooping cough, and tetanus (17, 18).

If we use fixation of the structures of some of the isolates that cause the disease, then inactivate them and preserve their whole structures, the possible deleterious effect of the high mutagenicity detected in a few of the proteins of the virus (19) would be overcome by the strong immune response generated against the whole virus structure. Therefore, the mutagenicity should not be critical for the generation of protection, and would not damage the efficacy of the whole vaccine. Furthermore, it might be that the whole virus inactivated vaccine could even induce some cross protection against other Coronovirus agents of SARS, which hold conservative structures (20).

Furthermore, whole inactivated vaccines are considered good candidates for designing universal vaccines capable of giving protection against multiple strains of Influenza virus (15).

It is true that an impressive amount of data about the DNA sequencing of the virus has been gathered in a relatively short period of time and with that, the knowledge of its biological properties increased enormously (23,927 sequences in PUBMED) (19, 21–26). However, for an urgent strategy, we could also take advantage of the lessons taught by the history of vaccinology in order to prevent the disease and save lives.

Furthermore, if we want to enhance the efficacy of the vaccine we should combine the inactivated virus with a good adjuvant. The adjuvant might contain saponins of *Quillaja saponaria* Molina, which induce antibodies of desired subtypes, promote both the cytotoxic antiviral CD8^+^ and CD4^+^ Th1 cell responses against the infection and that been used with success in vaccines against leishmaniasis (7, 27, 28), cancer (29), Malaria (30), Herpes zoster (31), and HIV (32).

In spite of the valuable guidelines from the work of Jenner and Pasteur, who with much fewer resources, developed vaccines that controlled and eradicated smallpox that showed a 40% mortality rate (33) and rabies with 100% mortality rate (34); and even with all the knowledge acquired since then, there is no urgent combined international effort to produce one unique vaccine. Instead, 6 months after the description of the first COVID-19 cases in China, 147 vaccines are reported to be in development all over the world and, only two of them contain the inactivated virus (35, 36). All the other formulations include live attenuated, non-replicating or replicating viral vector, recombinant protein, peptide base, virus-like particle, and virus DNA and RNA. Most of these vaccines are focused on only one antigen of the Coronavirus, therefore, these formulations will certainly be less potent than a vaccine made up of multiple antigens contained in the whole pathogen.

Furthermore, many of these formulations do not use the technology involved in any previously licensed vaccines (37). CEPI (Coalition for Epidemic Preparedness Innovation) estimated the development of Phase I clinical trials of 8 vaccines, Phase 2 and 3 trials for up to 6 vaccines and progression to regulatory approval and production of up to 3 candidates (38).

In fact, by May 11th, 2020 seven vaccines had already entered Phase I clinical trials: (1) encapsulated mRNA encoding protein S (Moderna and NIAID, USA); (2) Adenovirus expressing protein S (Cansino Biologics, China); (3) DCs modified with lentivirus expressing several proteins and CTLs (Shenzen Geno-Immune Medical, China); (4) an APC modified with lentivirus expressing several viral proteins (35); (5) Inno 4800, SARS CoV2 DNA Injection (Innovio, USA); (6) ChAdOx1 vaccine from the Jenner Institute, Oxford University, (UK) which is a genetically modified Adenovirus expressing Coronavirus proteins (39), and is also being tested in a Phase II trial; and finally (7) the whole inactivated coronavirus with Alum by Sinovac, China (40). Furthermore, on July 2nd, 2020 the WHO communicates that there are 18 COVID-19 candidate vaccines in clinical evaluation and more 129 under pre-clinical assays (36).

## Current Vaccines With Published Results of Preclinical Evaluation, Under Phase III Clinical Trials and Large-Scale Production

Only one of the vaccines under clinical trials is currently supported by a peer reviewed scientific publication in Science that was published on May 7th, 2020: the inactivated whole virus vaccine of Sinovac (40). The results of its pre-clinical assays in the mouse, rat and non-human primate model were published before, without peer review on April 13th in the bioRxiv. Later on, on May 13th, the results of the Chadox1 adenovirus vaccine of the Jenner Institute of Oxford University were published with no peer review in the bioRxiv (39). Until June 29th, there has been no peer reviewed publication of this vaccine.

Regarding the formulations, the inactivated whole virus Sinovac vaccine is composed of one isolate of Sars-CoV2 (CN2) obtained from a patient of China and Alum adjuvant (40), while the Chadox1 nCoV19 vaccine of Oxford is composed of a Chimpanzee recombinant adenovirus, which expresses the S protein of SARS-Cov2 (39).

Sinovac Biotech (China) in collaboration with several Universities, Public Health institutions and the Medical Academy of the Army of China have been able to produce a whole virus inactivated vaccine adjuvanted by alum that was stable and showed 99.8 to 100% sequence identity to 10 other isolates also obtained from broncheoalveolar fluid (BALF) of hospitalized patients (five in intensive care), from China, Italy, United Kingdom, Switzerland and Spain (40). The virus was propagated in cultures of Vero cells *in vitro* and inactivated with β*-*propiolactone (40). The use of Alum adjuvant is approved for human vaccines because it induces strong antibody responses, mainly of the IgG1 and IgE types that show efficacy against virus or bacterial diseases, which need neutralizing antibodies to be controlled. However, alum is a poor promoter of the cellular immune responses against pathogens (41).

In contrast, the Chadox1 nCoV-19 vaccine developed by Oxford University and AstraZeneca is composed of a recombinant non-replicant chimpanzee adenovirus, which expresses the S protein of Sars-CoV-2 (39). Different from the technology used for inactivated vaccines since the 1800's, this adenovirus platform was developed in 2012 (42). The authors aimed to include an adenovirus in the vaccine that would not infect humans, in order to avoid its potential rejection by human antibodies. The chosen Chimpanzee adenovirus was phylogenetically related to the human adenovirus. The inventors deleted the region E1 of the chimpanzee adenovirus genome in order to render the virus defective and non-replicant, while the E3 region was excluded to increase the insert capacity. In addition, a bacterial artificial chromosome (BAC) containing a codon-optimized full-length spike protein of SARS-CoV-2 with a human tPA leader sequence (39) was added between the deleted E1 region and E4 to facilitate the genetic modifications. This approach has been reported to improve genetic stability (42). However, additional modifications were needed to guarantee that the E4 region of the virus would express a human, instead of a simian protein, that would enable the virus recognition and propagation inside human cells in *in vitro* culture, for large-scale virus production (42).

Regarding the number of samples, the SINOVAC inactivated vaccine was tested in groups of 10 mice and 10 rats and in 4 cohorts of 10 monkeys (*Macaca mulatta*) (40), while the Chadox1 nCOV-19 vaccine was only tested in groups of 5–8 mice and in 6 Rhesus monkeys using only 3 monkeys as controls (Table 2) (39).

**Table 2:** Comparison of efficacies of vaccine candidates with published results.

**Results and end-points of efficacy**	**Whole virus inactivated vaccine (SINOVA)**	**Recombinant chimpanzee adenovirus expressing the S antigen (Chadox1 nCOV19)**
Sample size in mice	4 Groups of 10	5–8
Sample size in rats	4 Groups of 10	-
Sample size in monkeys	Groups of 4 and 4 groups of 10	6 Vaccinated x 3 controls
IgG anti-S antibodies in mice	High	High IgG2a, IgG2b, IgG1
IgG anti-RBD antibodies in mice	High	Nd
IgG anti-N antibodies in mice	Intermediate	Nd
Neutralizing antibodies in mice	High	3/5
Neutralizing antibodies in rats	High	Nd
Mice S-specific CD4^+^ and CD8^+^ Th1 T cells expressing	Nd	IFN-γ, TNF-α
Mice S-specific CD4^+^ and CD8^+^ Th2 T cells expressing	Nd	IL-4
Mice S-specific Th1 cytokines in supernatants	Nd	IFN-γ, TNF-α, IL-2
Mice S-specific Th2 cytokines in supernatants	Nd	IL-6
IgG anti-S antibodies in monkeys	High	High
IgG anti-RBD antibodies in monkeys	High	Nd
IgG anti-N antibodies in monkeys	Low	Nd
Virus neutralizing antibodies in monkeys	High	High
Th1 cytokines in sera of vaccinated monkeys	No variation of IFN-γ, TNF-α, IL-2	IFN-γ 6/6 and TNF-α in 1/6
Th2 cytokines in sera of vaccinated monkeys	No variation of IL-4, IL-5, IL-6	IL-6 1/6 and IL-10 in 1/6
CD3 lymphocytes	No variation	Nd
CD4 lymphocytes	No variation	Nd
CD8 lymphocytes	No variation	Nd
Cross protection to other SARS-CoV2 isolates	High	Nd
Viral load in nasopharynx	No	Yes
viral load in BALF	No	1/6
viral load in lungs	No	Partial
viral load in anal swabs	No	Nd
Safety in monkeys	Yes	Yes
Prevention against infection in monkeys	Yes	No
Prevention against disease in monkeys	Yes	No
Prevention against severe disease in monkeys	Yes	Yes
Prevention against mortality in monkeys	Nd	Nd

*Nd, not determined*.

Regarding the antibody response in mice and rats, the Sinovac vaccine promoted high IgG antibody titers against protein S, against its Receptor Binding domain, and to a lower extent against protein N (Table 2) and also high titers of virus neutralizing antibodies. The cytokine expression induced by the inactivated vaccine in mice was not analyzed (40). In contrast, the Chadox1 vaccine induced anti-S1 and S2 protein IgG antibody titers (IgG2a, IgG2b, and IgG1) and neutralizing antibodies in only three of the five BALB/c mice, but showed IFN-γ, TNF-α, and IL-4 expressed by CD4^+^ and CD8^+^ T cells and IFN-γ, TNF-α, IL-2, and remarkably IL-6 secreted to the supernatants (39) (Table 2).

Furthermore, in vaccinated monkeys, seven days after infection, the Sinovac inactivated vaccine at 6 μg/dose induced high titers of IgG antibodies directed against the S, RBD and lower levels of anti-N protein antibodies, high titers of virus neutralizing antibodies with no detected antibody-dependent enhancement of disease (ADE) (40). In contrast, anti-S protein IgG and neutralizing antibodies were detected in the 6 Rhesus monkeys vaccinated with Chadox1 (Table 2) (39). Moreover, regarding the concern of the increased pro-inflammatory events and cytokine storm related to the severity of COVID-19, the Sinovac vaccine was safe and did not promote any alteration in the frequencies of CD3^+^, CD4^+^, or CD8 T cells nor of secretion of IFN-γ, TNF-α, IL-2, IL-4, IL-5, or IL-6 (Table 2) (40). In contrast, increased levels of IFN-γ, TNF-α, IL-6, and IL-10 were observed in monkeys vaccinated with the Chadox1 vaccine (39) (Table 2) in which the frequencies of cytokine secreting T lymphocytes was not studied (Table 2).

Regarding cross-protection to other SARS CoV2 isolates, the Sinovac inactivated vaccine protected mice and rats against the challenge with 11 different virus isolates (Table 2) suggesting its potential use all over the World (40). There is no available data concerning cross-protection for the Chadox 1 vaccine (39).

Besides, for a fair comparison of vaccine efficacies, the two SARS-CoV2 vaccines should be assayed in the same field trial, and the efficacy end-points should be determined prior to the assay. Due to the urgency in saving lives, this might be not be feasible during the pandemic. However, for comparative purposes, early infection, disease, severe disease, and death due to COVID-19 or other causes should be recorded as vaccine efficacy end-points. For instance, reduction of the virus load in the nasal and pharynx mucosa indicates not only protection against early infection, but also the blockade of the transmission of infection by respiratory droplets. This means that this end point is particularly important when seeking a vaccine to interrupt the epidemic. In addition, clinical symptoms indicate disease, while pulmonary distress, cytokine storm, need of intensive care, intubation indicate severe disease. The number of deaths due to COVID-19 or other causes should also be recorded and compared in order to evaluate the reduction of mortality.

Notable, the Sinovac inactivated vaccine reduced to zero the viral load in throat swabs (pharynx and crissum), anal swabs and all regions of both lungs of vaccinated and challenged monkeys (40) indicating that the inactivated vaccine prevents not only the early infection but also blocks the transmission of the disease by droplets curtailing the epidemics. In contrast, no viral sgRNA indicative of viral replication, could be detected in BAL fluids, and in the lungs of two of six monkeys vaccinated with Chadox 1 and challenged (39) (Table 2). In fact, the lung viral load decreased by ~60% in monkeys vaccinated with Chadox1. However, viral gRNA was detected in nose swabs of all vaccinated and challenged animals (Table 2) (39), indicating that the Chadox1 vaccine would not prevent the SARS CoV2 human infection nor block its transmission and interrupt the epidemics. Vaccinated and infected subjects will continue to be infectious and spread SARS CoV-2. However, the vaccine will probably, in most cases, reduce the pulmonary symptoms, and make the disease less severe. Accordingly, the inventors of the Chadox1 vaccine seem to be aware of the limitations of its efficacy when they describe it as a vaccine that prevents pneumonia in monkeys (39).

Unfortunately, neither the investigations of the Sinovac nor the Chadox1 vaccine have disclosed if any of their formulations prevent or reduces mortality.

Furthermore, in a report that analyzes the first results of the vaccine trials, published in Nature, Peter Hotez considered that the Oxford vaccine induced very modest titers of neutralizing antibodies and that considerable higher titers would be needed to afford protection (43). At the same time, Hotez also says that the Sinovac vaccine elicited a more promising antibody response in macaques monkeys (43).

In spite of that, WHO disclosed that this vaccine is in fact being tested in UK in Phase I, II and III trials (36) and will be tested in a Phase III trial in Brazil on 2,000 volunteers. Consequently, contracts for large-scale fabrication have already been signed with the Public Laboratory of the Brazilian Ministry of Health Bio-Manguinhos. In Brazil, 30 million doses are intended to be produced by Bio-Manguinhos and another 100 million after the proven efficacy of the vaccine. At this point it is important to know which end-points of vaccine efficacy will be taken into consideration for such an important decision.

On the other hand, the Sinovac whole virus inactivated vaccine was also reported to have been successful in Phase I and II trials in 18–59 year olds (*n* = 422) and in healthy elderly adults >60 years old (*n* = 744) in China (36) although these results have not yet been published in detail. More than 90 % of the volunteers showed neutralizing antibodies (44). A recent contract has been signed between Sinovac and the public Laboratory Instituto Butantan of São Paulo, Brazil, in order to produce doses of the vaccine to immunize 8,870 healthcare professionals for a double-blind randomized Phase III trial in Brazil, where the incidence of cases and deaths due to COVID-19 is still high (45). The results of the efficacy are expected in October 2020. In return, the Instituto Butantan will gain the transfer of the technology and the license to manufacture 60,000,000 doses for Brazil. Testing anti-COVID-19 vaccines in Brazil became interesting because of the high morbidity and mortality and active expansion of the epidemics.

An important warning is given by Ewen Callaway in his article published in Nature (43), in which he asks for caution about the potential success of vaccines that arise from small animal or human studies. This might be the case of the Moderna-NIAID vaccine composed of lipid nano-particle encapsulated synthetic mRNA, which encodes the spike S protein and already underwent Phase I and II clinical trials in USA (36). Moderna company announced that Phase III trials are predicted to start in July 2020 and that studies in monkeys are underway in parallel. None of these mRNA based vaccine has ever been licensed before (43).

We conclude that the first results of anti-COVID-19 vaccine candidates confirm that the whole virus inactivated vaccine, which preserves the immunogenicity of all the antigens of the virus and contains PAMPS (40) is more potent than the recombinant vaccines that have only the important S spicula protein, either expressed by an engineered adenovirus (39) or by LNP encapsulated mRNA (43). Furthermore, the inactivated vaccine also contains the Alum adjuvant. There are other examples support the superiority of whole inactivated vaccines above those expressing recombinants single antigens. For instance, 7.5 μg/dose of the trivalent inactivated Influenza vaccine is as safe as, but more immunogenic than the 22.5 μg/dose of the recombinant baculovirus-expressed hemagglutinin FluBok vaccine, in young children (46).

This is a fast moving scenario and several Phase 1 clinical trials of COVID vaccines have been published, either with or without peer reviews. Two recombinant adenovirus vaccines expressing the S spike protein of SARS-CoV-2, the Chadox1 and the Cansino vaccines (47, 48), and two other vaccines composed of mRNA codifying for the S-protein (mRNA1273, Moderna vaccine) (49) or its RDB domain (mRNA BNT162b1 Pfizer-Biontech vaccine) (50, 51) have been assayed for safety and immunogenicity in Phase I-II clinical trials in humans. There were no serious adverse events related to any of the four vaccines (47–51). Local and systemic reactions commonly including pain, feeling feverish, chills, muscle ache, headache, and malaise were recorded for all formulations (47–51) and were reduced, in the case of the Chadox1 vaccine, with use of prophylactic paracetamol (47). Only the Cansino vaccine was given as a single dose (48) while Chadox1, Moderna, and Pfizer Biontech vaccines were assayed in two-dose protocols (47, 49–51). Anti-S protein IgG responses rose by day 14 (47) and peaked or increased by day 21–28, after the first (48) or second immunization dose, respectively (47, 49–51). In addition, spike-specific T-cell responses detected by an *ex-vivo* interferon-γ enzyme-linked immunospot assay, peaked on day 14 for the Chadox1 (47) and on day 28 for the Cansino adenovirus vaccine (48). Moreover, the Moderna mRNA-1,273 vaccine induced a Th1 response against the S-protein peptide pools (TNF-α >Il-2 >IFN-γ), with a minimal Th2 cytokine expression (IL-4 and IL-13) and with CD8 T-cell responses, only detected at low levels, after the second vaccination (49). In agreement, most participants vaccinated with the Pfizer-Biontech mRNA-RBD vaccine (BNT162b1) also had Th1 skewed T cell immune responses with RBD-specific CD8^+^ and CD4^+^ T cell expansion and IFN-γ produced by a high fraction of RBD-specific CD8+ and CD4+ T cells (51). Furthermore, the levels of neutralizing antibodies raised by each one of the vaccines could be considered as correlates of their potential efficacy. While the mRNA-1273 of Moderna disclosed 50% EC values ranging between 256 and 512 (49), the maximal titer for the mRNA RBD vaccine of Pfizer-Biontech was 308 (51) and for the Chadox1 vaccine, from 256 to 512 (47). The Cansino vaccine expresses its results as GMT (4–55, 61) impeding an accurate comparison (48). Unfortunately, the results of Phase I-II clinical trial of the whole virus inactivated vaccine of Sinovac have not yet been published in detail, therefore although the vaccine was tested in the largest number of individuals (*n* = 1,166) (36), a fair comparison of the safety and immunogenicity results is not yet possible. Ultimately, only the results of the Phase III trials will disclose the potential impact of the vaccines on reduction of deaths, clinical cases, and virus particles or viral RNA in nasopharynx and will allow their efficacy and capability to interrupt the epidemic to be evaluated.

## Discussion

In the imminence of a pandemic involving high mortality and economic distress, several factors could speed up the development of vaccines. One of them would be the use of an already standardized methodology. It is worth noting that most of the molecularly defined vaccines now in development would meet severe restrictions for large-scale production and this could led to an enormous delay to deliver vaccines for mass vaccination of the public. In contrast to this, nowadays large industries and public laboratories are authorized to produce inactivated vaccines against Influenza. It is also reasonable to hypothesize that generation of protection and immunological memory against a group of antigens will be more efficient than that generated against a single antigen, no matter, how important it is.

Two concerns could be considered as the downside of the inactivated vaccines for SARS diseases. The first would be the fear of an incomplete inactivation of the virus that could cause outbreaks among the vaccine production workers or in vaccinated populations (52). This concern is common to all vaccines produced with native antigens, which demand the production of large mass of pathogens. However, to guarantee safety, each batch of vaccine is submitted to validation of inactivation controls that include sequential passages assays of residual virus infectivity in embryonated eggs or tissue culture, and detection of live virus by TCID50 assays (14). The whole virus SARS-CoV-2 inactivated vaccine of Sinovac includes validation of inactivation controls (40). The second concern would be the promotion of an Antibody Disease Enhancement syndrome (ADE) by the vaccine. This is usually related to non-neutralizing antibodies, which determine an increased lung pathology and it was observed before in vaccines against RSV and Measles in the 1960's (53). Since SARS-CoV-1, MERS-CoV, and SARS-CoV-2 are phylogenetically related viruses that have caused epidemics over the last 16 years and ADE pathology was present for some SARSCoV-1 and MERS vaccine candidates in animal models, there is also a concern about the induction of ADE syndrome in humans vaccinated with SARS-CoV-2 vaccine candidates (53). However, ADE pathology is not exclusive for inactivated vaccines and has been also demonstrated for the vectored vaccine expressing N protein, a replicon particle platform expressing S protein (53), the recombinant protein S with or without gold nanoparticles (54) and a MVA vectored vaccine expressing S proteins (53, 55).

With the aim of preventing these safety issues in SARS-CoV-2 vaccines CEPI and the Brighton Collaboration Safety Platform for Emergency Vaccines (SPEAC) convoked an expert scientific meeting on March 12 and 13, 2020 in order to establish the assessment of the risk of ADE during SARS-CoV-2 vaccine development (53).

In murine models, ADE was observed for an inactivated whole virus vaccine against MERS (56) and against SARS-CoV-1 (53, 57, 58). In fact an inactivated MERS-CoV vaccine, with and without adjuvant, induced in mice neutralizing antibody, reduced the viral load in lungs but showed mononuclear infiltrates containing eosinophils and eosinophils secreting IL-5 and IL-13 cytokines (56). A formalin double-inactivated SARS-CoV-1 (DIV) vaccine, adjuvanted or not, induced also immunopathology involving eosinophils in aged mice (53, 57). In addition, a formalin-inactivated SARS-CoV-1 vaccine promoted ADE in NHP with macrophage and lymphocyte infiltration in the lungs and fibrin and protein-rich edema in the alveolar cavity (58). On the other hand, other inactivated vaccines against SARS were reported as non-inducers of ADE (59).

Fortunately, other studies disclosed the absence of ADE in hamsters and monkeys immunized with whole inactivated vaccine against SARS-CoV-1. These studies differed from the previous one in the use of β-Propiolactone instead of formalin to inactivate the virus (60, 61).

The conclusion was that, NHPs could be used to evaluate the anti-COVID-19 vaccines with or without adjuvants to select the formulations with desired efficacy and reduced risk of ADE (53). In addition, transgenic mice expressing the human ACE receptor will be needed to evaluate the vaccine induced ADE. The immunopathology was a consequence to a Th2 type of response to the antigen and it was avoided in vaccines that drive the response to a Th1 immunity, with or without adjuvants. Also, it is known that the presence of fetal calf serum in the preclinical vaccine preparation may induce eosinophil influx to lungs (53).

For instance, the passive transfer in NHPs of human antibodies generated during Phase 1 trials, followed by viral challenge could be considered to assess the risk of disease enhancement (53). It was recommended to challenge the vaccinated animals with close related species in order to evaluate cross protection for future epidemic (15, 53). This has been done for the whole inactivated Sinovac vaccine with other isolates of the SARS-CoV-2 (40). Experts also recommended including animals vaccinated with formalin inactivated, alum-adjuvanted whole virus SARS-CoV-1 or SARS-CoV-2, for immunopathology studies, as positive controls. This will help in establish accepted end-points to allow comparison (53). The group of experts considers that continuous monitoring of this risk will be needed during clinical trials. Each effect observed should be discussed by the developers with their regulators who will ultimately define the actual requirements for clinical studies (53).

If we consider that a whole virus inactivated vaccine with a potent QS21 saponin adjuvant is the ideal formulation for an anti-COVID-19 urgent first vaccine, the Sinovac vaccine is not only the closest formulation to the ideal, only differing in the adjuvant, but also the one that can be developed the fastest. The potential use of alum, MF59, AS03, AS04, or AS01, which contain QS21 saponin has been discussed (53, 62). It was concluded that, the immunopathology of SARS vaccines was a consequence to a Th2 type of response to the antigen and it was avoided in vaccines that drive the response to a Th1 immunity, with or without adjuvants (53).

Time matters and is an extremely important factor considering the high daily rate of deaths worldwide. In fact, the assays of the Sinovac vaccine in the mouse, rat, and macaque models seems to have been performed simultaneously from January to March, 2020. In addition, this is the only vaccine with results already published in a peer reviewed Journal (40).

On the other hand, eradication of the pandemic or at least its control, as Jenner knew in 1796, will only be possible by a universal and simultaneous use of the same vaccine. This is what took place with smallpox, rabies, yellow fever, Influenza, H1N1, etc. In spite of that, we do not see the united international effort to gather together resources for the production of enough doses of one vaccine to vaccinate the World. The support given to 147 different research projects (36) and the deposit of hundreds of patents confirms that. Again, the urgent formulation might already be known and waiting to be rediscovered from the history of vaccinology. If different vaccines with diverse degrees of efficacy values are used, even the countries that have low incidence of COVID-19 will not be safe and will not be able to open their frontiers. To support the production of one ideal vaccine should be the common focus worldwide.

Maybe the observed multiple individualistic efforts that have arisen are due to the lack of leadership from the developed nations, which have the highest capacity to produce vaccines. In the USA, for instance, the economic interest of the large vaccine industries in preventive vaccination has recently decreased. Conversely, they have started to invest in immunotherapies or drug treatments. In addition, in the USA there are not large Public Laboratories for production of the vaccines under a governmental request. Consequently, the governmental Public Health decisions are restricted by the interests of the private vaccine companies. In contrast, in some developing countries, where infectious diseases are often the most important causes of mortality, Public Laboratories can produce large amounts of vaccine doses without the need to make a profit, under the auspices of their Ministries of Health. This is the case of Instituto Butantan and Bio-Manguinhos in Brazil, Instituto Biológico de La Plata and the Administración Nacional de Laboratorios e Institutos de la Salud (ANLIS-Malbrán) in Buenos Aires, Argentina, and of the Serum Institute of India. Fortunately, Instituto Butantan will produce the Sinovac inactivated vaccine and Bio-Manguinhos the adenovirus Chadox1 vaccine of Oxford in Brazil. The Serum Institute of India will also produce the Chadox1 vaccine of Oxford.

Finally, the modern policies for vaccine regulations should be taken into consideration. These regulations demand that Phase I, Phase II, and Phase III trials should be developed with success before a government licenses a vaccine and uses it on Phase IV trials and for industrialization. This usually takes at least a decade. Although these tests enhance the confidence of a product, one might think that if we are dealing with a vaccine produced by a technology that is already well-established in many licensed vaccines, such as a whole inactivated virus, more rapid or simultaneous tests would be accepted as proofs of concepts (38). This would be another increased cost-benefit value of the vaccine, which takes into account the high mortality, worldwide incidence and impressive impact on the economy promoted by the quarantines. Probably, this is what Jenner and Pasteur would have done.

## Author Contributions

CP-S designed and wrote this article.

## Conflict of Interest

The author declares that the research was conducted in the absence of any commercial or financial relationships that could be construed as a potential conflict of interest.
